# Towards a gene regulatory network shaping the fins of the Princess cichlid

**DOI:** 10.1038/s41598-018-27977-y

**Published:** 2018-06-25

**Authors:** Ehsan Pashay Ahi, Kristina M. Sefc

**Affiliations:** Institute of Biology, University of Graz, Universitätsplatz 2, A-8010 Graz, Austria

## Abstract

Variation in fin shape and size contributes to the outstanding morphological diversity of teleost fishes, but the regulation of fin growth has not yet been studied extensively outside the zebrafish model. A previous gene expression study addressing the ornamental elongations of unpaired fins in the African cichlid fish *Neolamprologus brichardi* identified three genes (*cx43*, *mmp9* and *sema3d*) with strong and consistent expression differences between short and elongated fin regions. Remarkably, the expression patterns of these genes were not consistent with inferences on their regulatory interactions in zebrafish. Here, we identify a gene expression network (GRN) comprising *cx43*, *mmp9*, and possibly also *sema3d* by a stepwise approach of identifying co-expression modules and predicting their upstream regulators. Among the transcription factors (TFs) predicted as potential upstream regulators of 11 co-expressed genes, six TFs (*foxc1*, *foxp1*, *foxd3, myc, egr2, irf8*) showed expression patterns consistent with their cooperative transcriptional regulation of the gene network. Some of these TFs have already been implicated in teleost fish fin regeneration and formation. We particularly discuss the potential function of *foxd3* as driver of the network and its role in the unexpected gene expression correlations observed in *N. brichardi*.

## Introduction

The developmental mechanisms underlying fin formation in fish display remarkable similarities with those involved in appendage development in other vertebrates^1–3^. The ability of teleost fish to completely regenerate amputated fins from adult, differentiated cells provides a fascinating opportunity to investigate the molecular mechanisms of tissue regeneration in higher vertebrates^4^. Buoyed by the availability of mutants for teleost models (primarily zebrafish), studies of fin morphogenesis have elucidated molecular details about underlying genetic factors and signaling pathways^5^. In comparison, however, much less is known about the genetic factors involved in the natural morphological variation of fin shape in teleost fishes.

The fin of teleost fish is comprised of bifurcated structures, termed fin rays or lepidotrichia, which include vascularized and innervated mesenchyme enclosed by bony segments and several epidermal layers. The fin rays are connected by inter-ray tissue which also contains mesenchyme surrounded by epidermis. The fin growth is the result of the distal addition of segments through cell proliferation, differentiation and survival^6^. A partially amputated fin is capable of regeneration through the formation of a highly proliferative tissue (blastema) at the distal end of rays and inter-ray tissue. Despite the simple structural properties, fin regeneration involves complex processes of dedifferentiation of cells into blastema, proliferation of undifferentiated blastemal cells towards the distal end and differentiation of blastemal cells in the proximal end of the regenerating tissue^1,7^. At the molecular level, both ontogenetic and regenerative fin growth are tightly regulated by several interconnected signaling pathways and their downstream effectors^6–8^.

**Figure 1:**
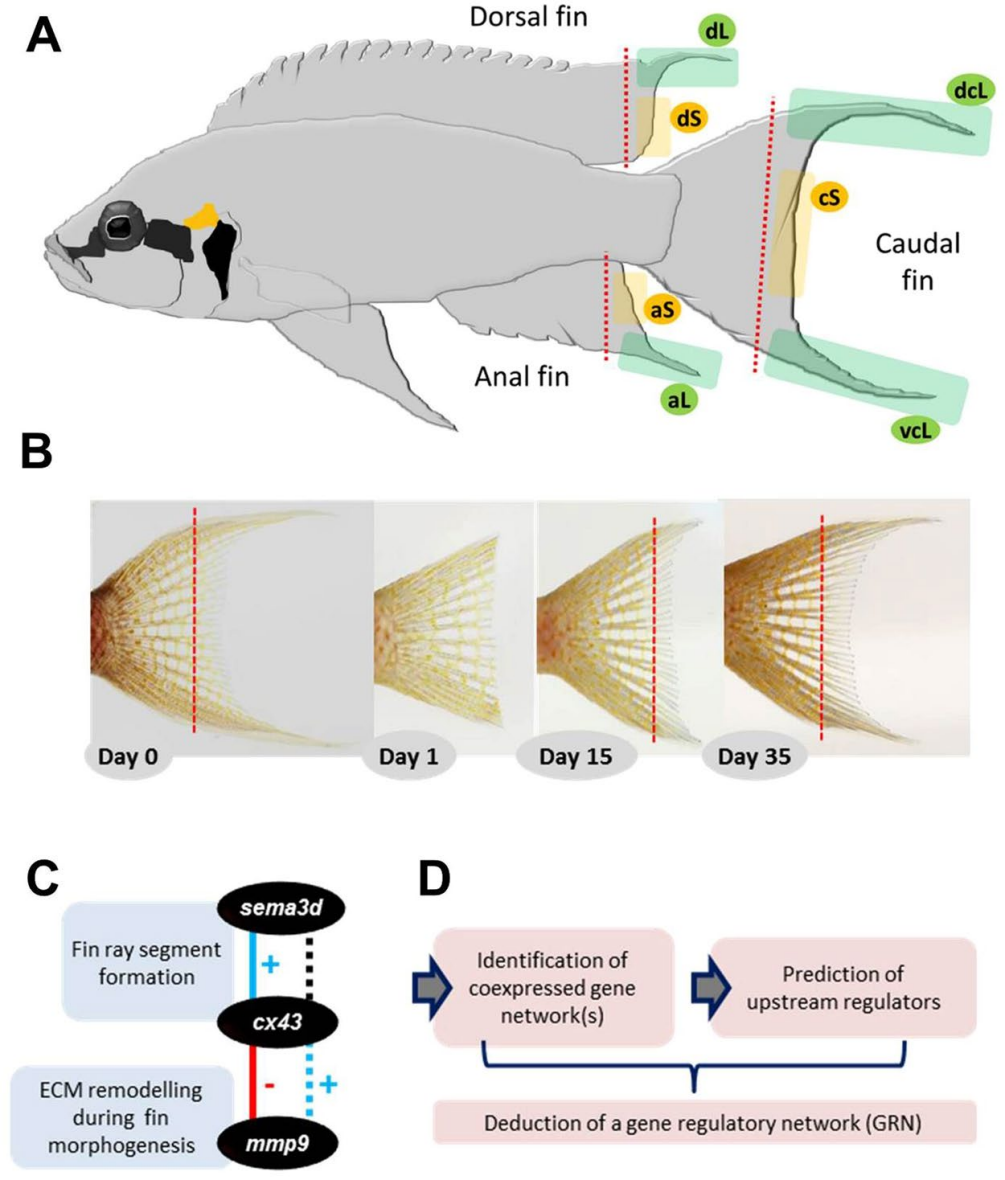
Fin dissections and a workflow for identifying gene regulatory network(s) underlying elongated fin phenotype in *Neolamprologus brichardi*. (**A**) An adult Lake Tanganyika cichlid fish, *N. brichardi*, displays filamentous elongations of the unpaired fins. Green shaded areas mark the elongated regions of the dorsal fin (dL), anal fin (aL), and the ventral and dorsal regions of the caudal fin (dcL and vcL); yellow shaded areas mark the short regions in the dorsal fin (dS), anal fin (aS), and in the center of the caudal fin (cS). The red dashed line represents the cutting line for the biopsy. (**B**) Tissue sampling at day 0 (first cut), day 15 (second cut) and day 35 (third cut) using the example of the caudal fin (photos by Wolfgang Gessl (www.pisces.at)). (**C**) Genes found to be associated with the elongated fin phenotype of *N. brichardi*, their function in zebrafish (blue squares) and their positive or negative expression correlation in zebrafish (continuous blue and red lines, respectively), as well as their contrasting expression correlation in *N. brichardi* (dashed lines; black colour indicates no expression correlation). (**D**) Schematic representation of the steps involved in the deduction of the gene regulatory network based on zebrafish co-expression data for *sema3d*, *cx43* and *mmp9*.

Extensive research has been launched to identify genes underlying fin growth and regeneration with a strong focus on the caudal fin of the zebrafish model *Danio rerio*^2,8–10^. It is only in recent years that the molecular basis of the morphological diversity of fins within and across species has attracted some attention^11–15^. Studies capitalizing on the natural variation in fin morphology addressed, for instance, the ventral elongation of the caudal fin in swordtail fish^11^, interspecific divergence in pectoral fin morphology in cichlids from Lake Malawi^13^ and the twin-tail phenotype of goldfish^14^. Here, we are interested in the molecular basis of fin filaments, that is, ornamental elongations of fins which are displayed by numerous fish species across various taxonomic groups. In our study species, the African cichlid fish *N. brichardi*, the unpaired fins of both males and females are conspicuously adorned by such filamentous elongations (Fig. 1A). In a previous study, we hypothesized that positional differences in gene expression levels underlie the extreme elongation of filaments, and tested a series of candidate genes involved in fin formation and regeneration for differential expression between elongated (L) and regular (i.e. short, S) fin regions^15^. Comparing gene expression levels between L and S tissue sampled from both intact and regenerating fins, we detected several genes with either higher L-expression or higher S-expression^15^. Particularly strong and consistent signals were obtained for *cx43/ Gja1* and *mmp9*, both showing elevated L-expression, and *sema3d* with elevated S-expression. *cx43* encodes for a subunit of the gap junction protein complex^16^, *mmp9* produces a matrix remodelling enzyme^8,17^ and *sema3d* encodes a conserved secreted ligand of several cell surface receptors involved in nervous system development, cell differentiation and bone homeostasis^18,19^. In zebrafish, *sema3d* functions downstream of *cx43* in a common pathway regulating cell proliferation and joint formation, since a knockdown of *cx43* results in reduced expression of *sema3d*^18^. Also in zebrafish, reduced expression of *cx43* was associated with up-regulation of *mmp9* in the caudal fin^20^. Conversely, in *N. brichardi*, we found no correlation between *cx43* and *sema3d* expression, whereas the expression of *cx43* was positively correlated with *mmp9* expression in each of the unpaired fins^15^. We also found *sema3d* expression to be positively correlated with the expression of a ligand of Wnt signaling pathway (*wnt5b*) across all fins, which suggested its transcriptional regulation by this pathway, consistent with findings in zebrafish neural crest cells^21^. Interestingly, the elevated expression of *cx43* in the elongated fin regions of *N. brichardi* was not accompanied by an increase of the length of the fin ray segments, which contrasts with the effects of *cx43* manipulation in zebrafish^16,22^. A recent study, however, suggests diverse functions of *cx43* during zebrafish skeletal growth and different mutants of *cx43* can confer distinct bone phenotypes in fin and vertebrae^23^, though the underlying gene regulatory networks have yet to be elucidated.

The expression patterns observed in our previous study raise the possibility that in the unpaired fins of *N. brichardi*, *mmp9* and *cx43* belong to a co-expression network regulated by shared upstream player(s). It is worth emphasizing that the inter-dependent functional modulation of cx43 and mmp enzymes and their coexpression have attracted considerable attention due to the high abundance and diverse functions of these enzymes in various tissues^24^. However, little is known about their transcriptional co-regulation, particularly in the context of skeletal morphogenesis. The lack of an expression correlation between *sema3d* and *cx43* suggests their regulatory decoupling and the potential involvement of a distinct upstream effector for *sema3d* during fin morphogenesis in *N. brichardi*, in contrast to evidence from zebrafish^18^. Alternatively, *sema3d* might pertain to the same regulatory network as *cx43* and *mmp9*, but its expression might be repressed by certain regulator(s) of the network. In this study we sought to identify gene regulatory networks (GRN) comprising *cx43*, *mmp9*, and *sema3d* using a stepwise approach of identifying co-expression module(s) and predicting their upstream regulators^25^ (Fig. 1D). Candidate genes were identified from co-expression data available for zebrafish and tested for co-expression with *mmp9*, *cx43* and *sema3d* in the intact and regenerating fin tissue of *N. brichardi*. The prediction of transcription factors for genes of interest was based on the annotated genome of the Nile tilapia. Among the transcription factors (TFs) predicted as potential upstream regulators of 11 co-expressed genes, six TFs showed an expression pattern consistent with their cooperative transcriptional regulation of the gene network. In particular, one of the TFs, *foxd3*, may underlie the observed expression patterns of *cx43*, *mmp9* and *sema3d* in *N. brichardi*. Thus, we provide the first evidence for a potential GRN comprising *cx43*, *mmp9* and *sema3d*, as well as several other genes with unknown roles in fin formation, and provide a basis for further functional investigations in model and non-model organisms.

## Results

### Expression analysis of candidate genes co-expressed with *mmp9*, *cx43* and *sema3d*

**Figure 2:**
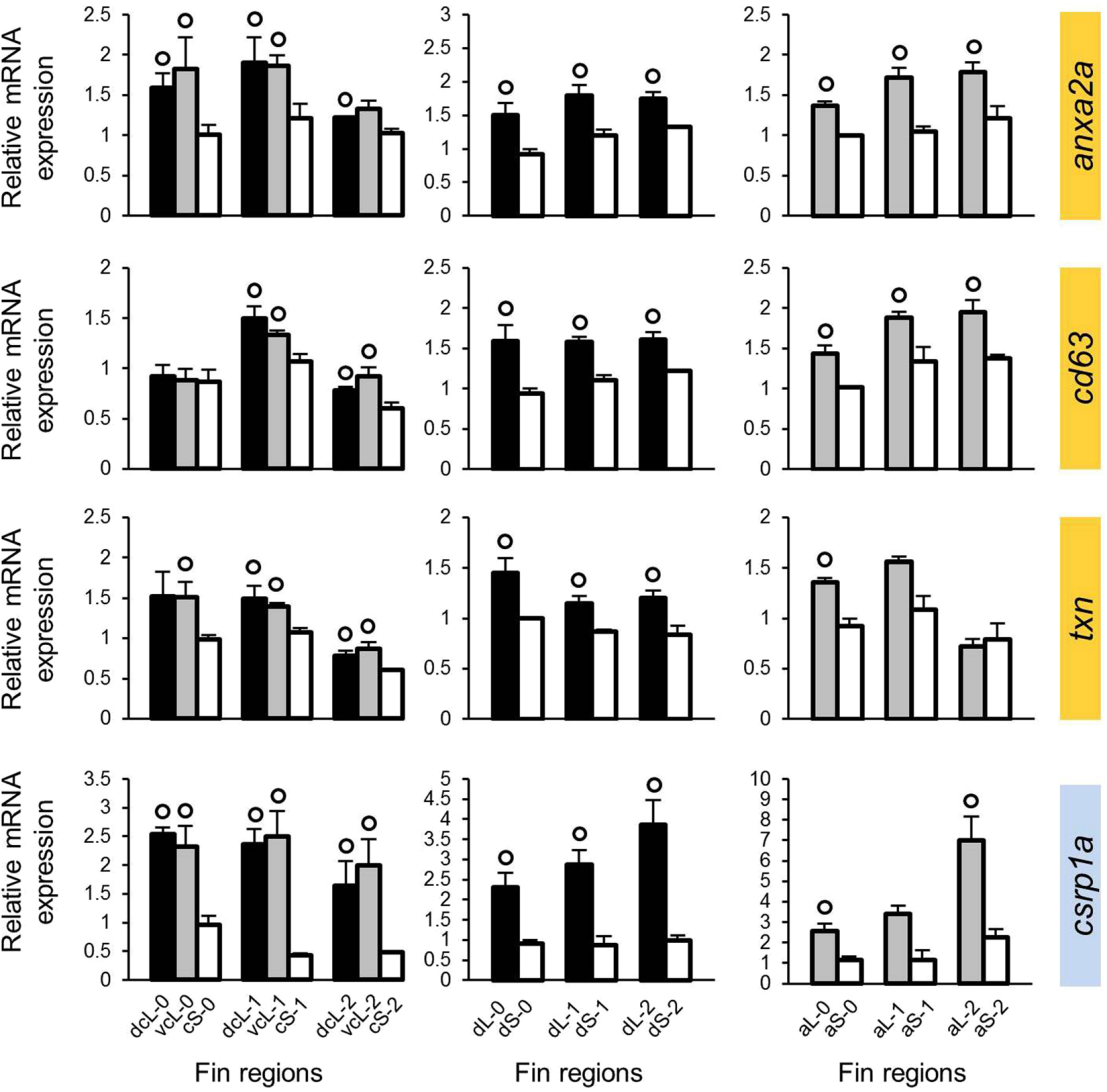
Expression levels of candidate genes selected based on co-expression with *mmp9*. Means and standard deviations of RQ in three biological replicates are shown for the elongated (L) and short (S) regions of the caudal, dorsal and anal fin in original (stage 0) and regenerating tissue. See Fig. 1A for fin region codes; numbers 0 to 2 identify regeneration stages. Circles above bars indicate significantly elevated expression (P < 0.05 in paired t-tests) in comparisons between L and S tissue samples (i.e., compared to the bar matching the shade of the circle); note that the analysis was restricted to comparisons within the same fin type and the same regeneration stage. Genes highlighted in yellow and blue were identified in the first and second step of our gene selection procedure, respectively.

**Figure 3:**
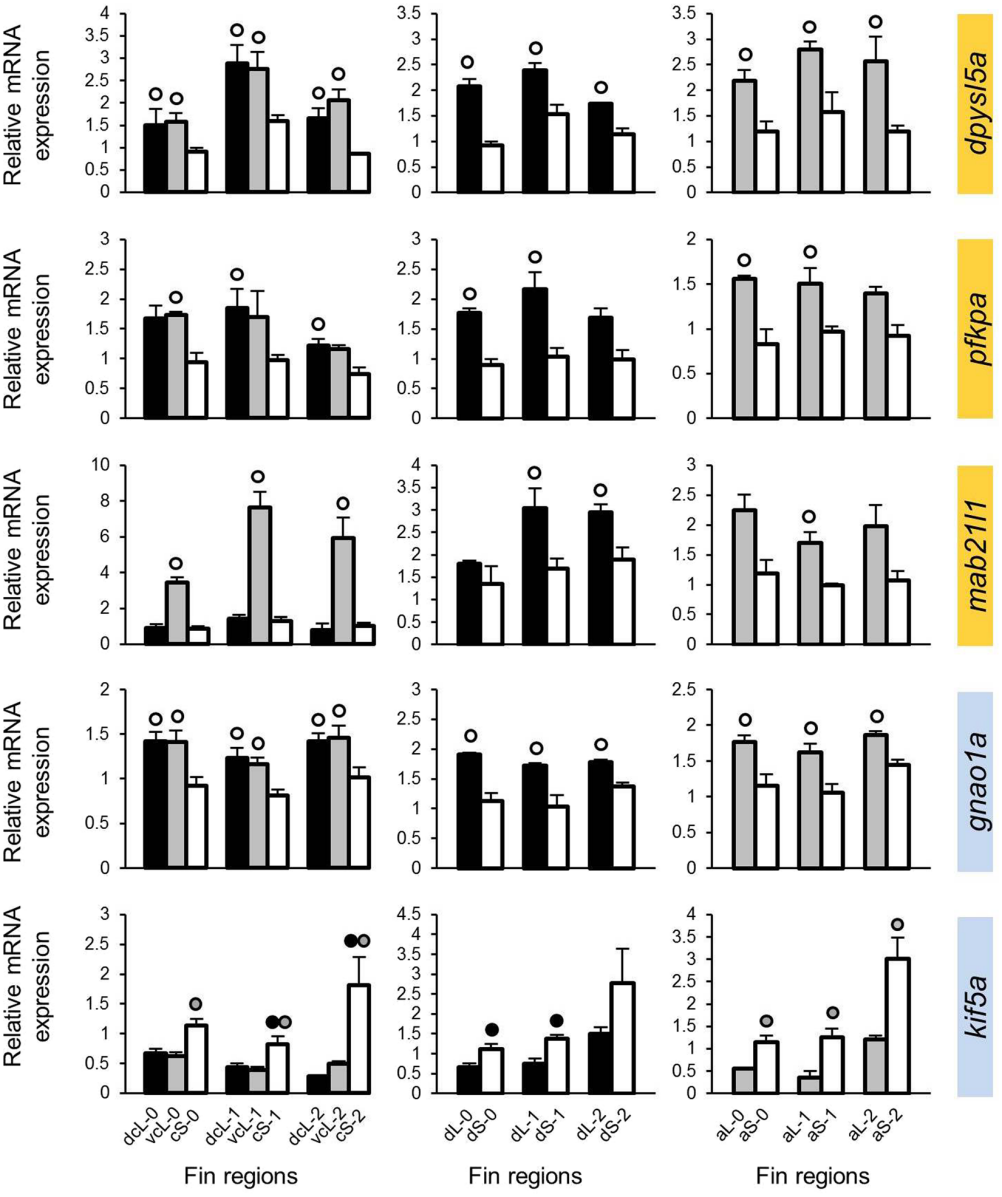
Expression levels of candidate genes selected based on co-expression with *cx43*. Means and standard deviations of RQ in three biological replicates are shown for the elongated (L) and short (S) regions of the caudal, dorsal and anal fin in original (stage 0) and regenerating tissue. See Fig. 1A for fin region codes; numbers 0 to 2 identify regeneration stages. Circles above bars indicate significantly elevated expression (P < 0.05 in paired t-tests) in comparisons between L and S tissue samples (i.e., compared to the bar matching the shade of the circle); note that the analysis was restricted to comparisons within the same fin type and the same regeneration stage. Genes highlighted in yellow and blue were identified in the first and second step of our gene selection procedure, respectively.

**Figure 4:**
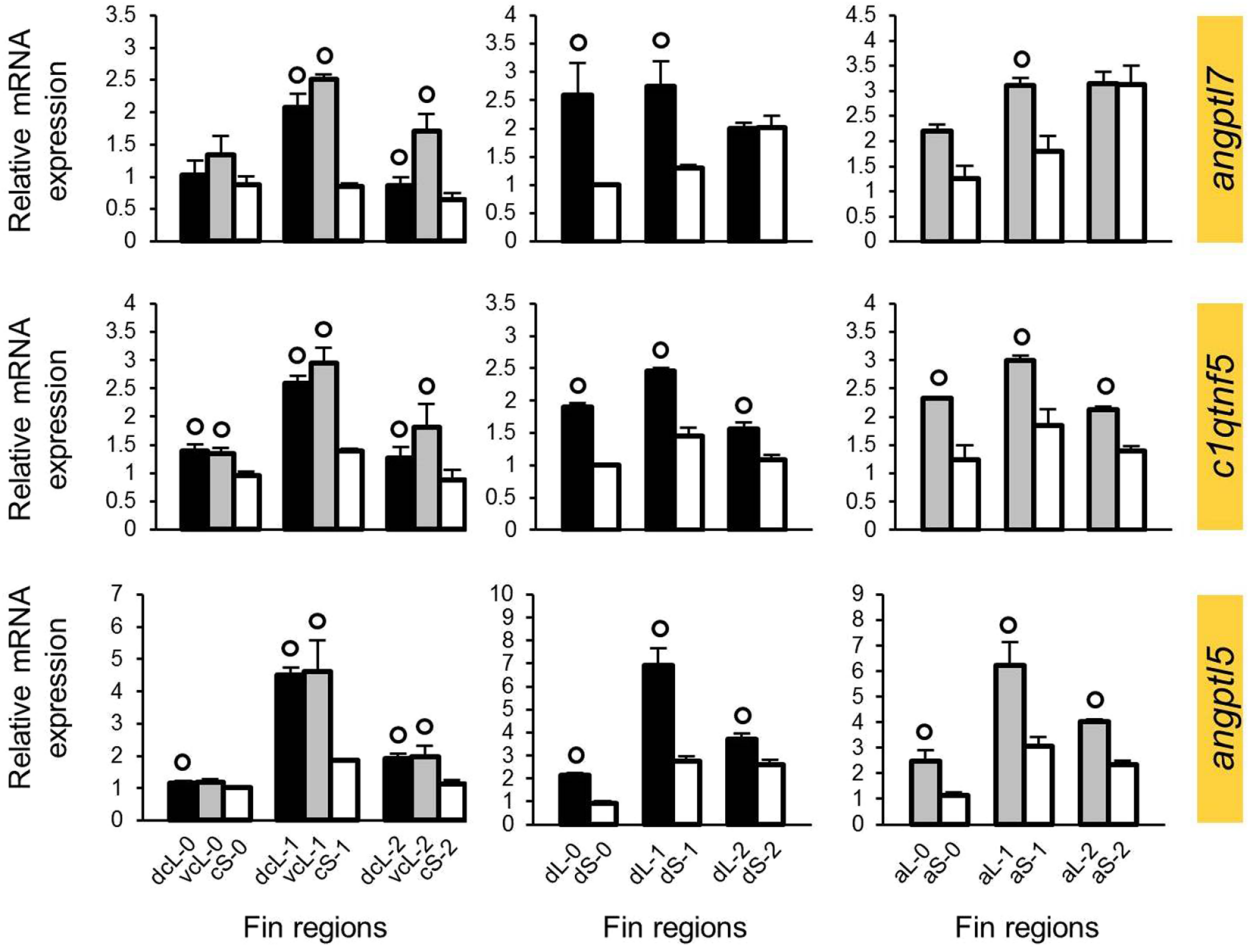
Expression levels of candidate genes selected based on co-expression with *sema3d*. Means and standard deviations of RQ in three biological replicates are shown for the elongated (L) and short (S) regions of the caudal, dorsal and anal fin in original (stage 0) and regenerating tissue. See Fig. 1A for fin region codes; numbers 0 to 2 identify regeneration stages. Circles above bars indicate significantly elevated expression (P < 0.05 in paired t-tests) in comparisons between L and S tissue samples (i.e., compared to the bar matching the shade of the circle); note that the analysis was restricted to comparisons within the same fin type and the same regeneration stage. Genes highlighted in yellow and blue were identified in the first and second step of our gene selection procedure, respectively.

In order to identify gene co-regulatory network(s) involved in the outgrowth of filaments on the unpaired fin of *N. brichardi*, we conducted stepwise candidate gene selection (described by Ahi *et al*.^25^) using a zebrafish co-expression database, COXPRESdb^26^. Our previous study suggested the genes *mmp9*, *cx43* and *sema3d* to be associated with the fin phenotype, based on expression differences between elongated and short fin tissue^15^. For each of these genes, we selected 8–9 strongly co-expressed genes from the zebrafish database (Supplementary data 1). Two of these genes, *bmp4* and *junb*, had already been included as candidate genes in the previous study^15^, such that 23 new candidate genes were inferred at this step. We tested the expression of these genes in the intact fin tissue (stage 0) and at two stages during regeneration (stages 1 and 2). In the following text ‘expression in the elongated region’ is abbreviated as ‘L-expression’, and reported as ‘higher’ or ‘lower’ in comparison to expression in the short region (‘S-expression’). In our previous study, L-expression of *mmp9* and *cx43* was higher and L-expression of *sema3d* was lower than their respective S-expression^15^. For 14 of the 23 tested genes, linear mixed models detected significant L/S expression differences (after Bonferroni correction for multiple testing), some of which were confounded by significant interactions with fin type and developmental stage (Supplementary data 2). Based on post-hoc tests (paired t-tests) used to identify L/S differences that were replicated across fin types and across developmental stages, we then identified 9 genes (3 genes for each set of the candidates co-expressed with *mmp9*, *cx43* and *sema3d*) with significant L/S expression differences in at least two stages of at least two fins (genes marked yellow in Figs 2–4; Supplementary data 2).

All of these genes had higher L-expression, although not necessarily in each fin or each regeneration stage (e.g. *mab21l1*, *txn* and *angptl5*; Figs 2–4). Noteworthy, the expression correlations of the 9 genes with L/S expression differences and the genes on the basis of which they were selected for the analysis (i.e., *mmp9*, *cx43* or *sema3d*) were positive in the zebrafish database. Therefore, higher L-expression was expected for genes associated with *mmp9* and *cx43*, whereas the genes selected on the basis of their co-expression with *sema3d* in zebrafish were expected to behave in the opposite way (*i.e*. lower L-expression, consistent with *sema3d*). Indeed, the 6 genes co-expressed with *mmp9* and *cx43* displayed higher L-expression (genes marked yellow in Figs 2 and 3), but opposed to expectations based on zebrafish expression data, the 3 genes co-expressed with *sema3d* showed opposite L/S expression differences to *sema3d* (i.e. again higher L-expression).

Based on the above data, we combined those genes, which showed consistent L/S expression patterns (i.e. consistent higher L-expression), into modules (module 1: *mmp9*-*anxa2a*-*cd63*-*txn*, module 2: *cx43*-*dpysl5a*-*pfkpa*, and module 3: *angptl7*-*c1qtnf5*-*angptl5*; *mab21l1* was ignored due to its inconsistent expression pattern), which were used to select additional candidate genes in order to extend our potential network. For each module, we selected five genes which were co-expressed with all the genes in the module according to the zebrafish database (Supplementary data 1), and tested them for L/S expression differences in the *N. brichardi* fins as described above (Supplementary data 2). This led to the identification of three more genes with L/S expression differences in almost all stages and fins derived from the first and second module (genes marked blue in Figs 2 and 3), whereas no L/S expression difference was detected in the genes derived from the third module. Two genes, *csrp1a* and *gnao1a*, had higher L-expression, and *kif5a* showed lower L-expression. We also note that the expression of *gnao1a* was very stable across regeneration stages (Fig. 3; Supplementary data 2). After the two steps of gene selection described above, a total of 10 genes (8 in the first step, 2 in the second step) were found to have consistently higher L-expression, as previously found for *cx43* and *mmp9*. Positive expression correlations in most pairwise comparisons among these genes suggested that they could pertain to a co-regulated gene network with shared upstream transcriptional regulators.

### Prediction of upstream regulators

**Figure 5:**
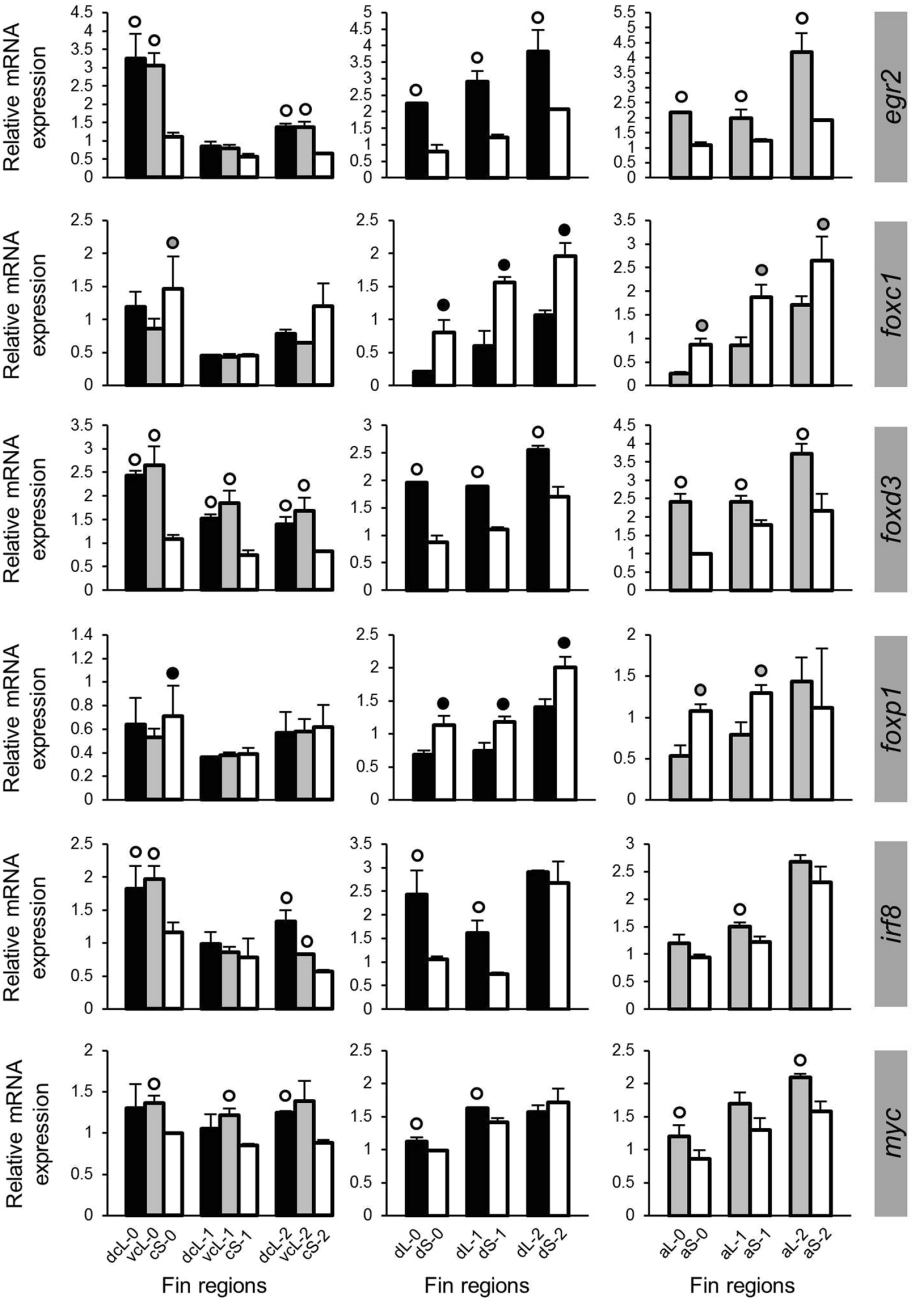
Expression levels of predicted upstream regulators. Means and standard deviations of RQ in three biological replicates are shown for the elongated (L) and short (S) regions of the caudal, dorsal and anal fin in original (stage 0) and regenerating tissue. See Fig. 1A for fin region codes; numbers 0 to 2 identify regeneration stages. Circles above bars indicate significantly elevated expression (P < 0.05 in paired t-tests) in comparisons between L and S tissue samples (i.e., compared to the bar matching the shade of the circle); note that the analysis was restricted to comparisons within the same fin type and the same regeneration stage.

In order to maximize the power of our approach, the following step was based on genes with congruent L/S expression differences (i.e., higher L-expression). We searched for potential upstream regulators of the identified gene network through prediction of TF binding sites in the upstream regulatory sequences of *cx43*, *mmp9* and the 10 new genes with consistently higher L-expression (i.e. *mab21l1* was dropped due to its inconsistent expression pattern; *sema3d* and *kif5a* because of their higher S-expression). We found more than 30 motifs present in the regulatory sequences of at least half of the genes (Supplementary data 1). By parsing the motifs against the vertebrate TF binding sites, we compiled a list of top matched TFs for each motif (Supplementary data 1). After analysing the expression levels of the 13 most significantly enriched TFs predicted by two different algorithms in the fins of *N. brichardi*, we found six TFs displaying L/S differential expression (Fig. 5; Supplementary data 2). Two of these TFs, *foxc1* and *foxp1*, showed lower L-expression, whereas the others showed higher L-expression. The most consistent differential expression was observed in *foxd3*, with higher L-expression across all fins and stages. Finally, we checked whether these 6 TFs had binding sites in the upstream regions of *sema3d* and *kif5a*, i.e. the two candidate genes with S > L expression. Indeed, binding sites for *foxd3*, *foxc1* and *irf8* are present upstream of *sema3d*, and *irf8* also has a binding site upstream of *kif5a*. Taken together, these findings implicate the involvement of several TFs in regulating the co-expression network through potential cooperative interaction(s) and short distance cis-binding promoter activity. Furthermore, *foxd3* appeared to be an upstream candidate for transcriptional induction of *cx43* and *mmp9* while possibly acting as a transcriptional repressor of *sema3d* in *N. brichardi* fins.

### Expression correlations

**Figure 6:**
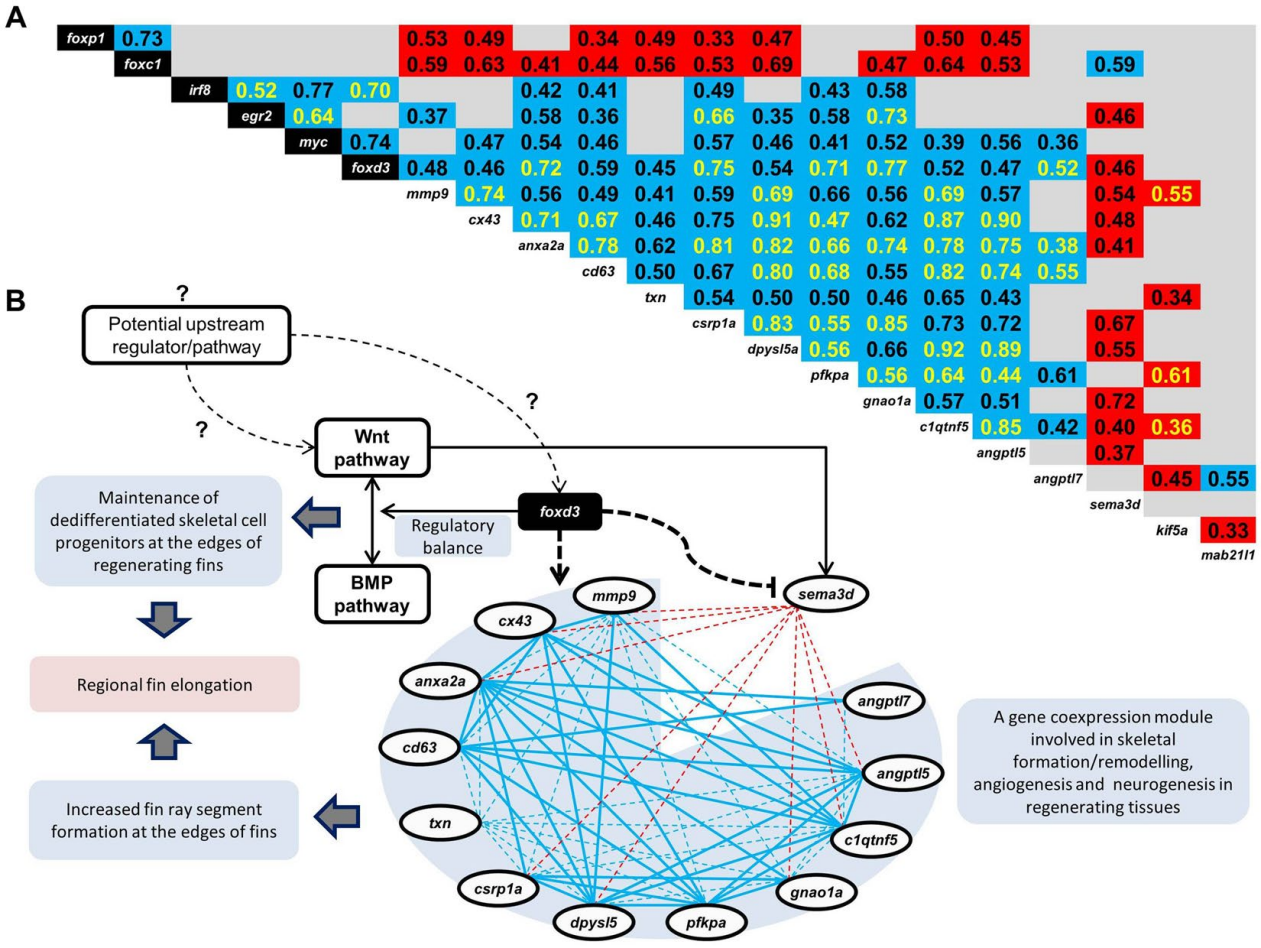
A proposed gene regulatory network underlying fin shape elongation in *Neolamprologus brichardi*. (**A**) Significant expression correlations between members of a gene network and their predicted upstream regulators across the unpaired fins of *N. brichardi*. Numbers indicate Pearson correlation coefficients (P < 0.01 in 2-tailed tests) based on gene expression data pooled across fins. Blue and red shadings represent positive and negative expression correlations, respectively. Yellow font indicates expression correlations, which were also significant in each of the three fins when analysed separately. (**B**) A proposed gene interaction model linking the identified genes and their functions, as well as their role in fin morphogenesis and regeneration in *N. brichardi*. In the co-expression module, positive (blue lines) and negative (red lines) expression correlations are indicated by solid lines, if the correlation was detected in each of the unpaired fins, while fin-specific correlations are indicated by dashed lines. Previously described regulatory connections are represented by black solid lines. Potential transcriptional induction and repression inferred in the present study is depicted by dashed black lines, and questionmarks indicate potential upstream regulatory connections which are not investigated in this study.

Pairwise expression correlation analyses among the investigated genes, *i.e*. TFs and the candidate network genes, used data pooled across all fins (Fig. 6) as well as for each fin separately (Fig. S1). Almost all genes of the putative co-expression network (except *angptl7*) showed positive expression correlations with rest of the members in data pooled across fins (blue shadings in Fig. 6A). *sema3d* displayed negative expression correlations with most members of the network and *kif5a* showed negative correlations with five members (red shadings in Fig. 6A). Some of the expression correlations between the genes (mainly positive ones) were detected in each of the fins (yellow numbers in Fig. 6A).

TFs varied in the number of co-expressed network genes, which suggests that some TFs participate in the regulation of more network genes than others. In particular, *foxc1* and *foxp1*each showed a high number of negative correlations, indicating a potential repressive regulatory role of *foxc1* and *foxp1* on the transcription of the gene network. Interestingly, *foxc1* had a positive expression correlation with *sema3d*. The remaining TFs, most notably *foxd3* and *myc*, had positive correlations with numerous network genes, suggesting a potential inductive regulatory role of these TFs on transcription of the gene network. Again, correlations of TFs with *sema3d* were in the opposite direction, i.e. negative (*egr2* and *foxd3*).

Many of the expression correlations between TFs and the network genes, which were detected when data were pooled across all fins, were not observed in each of the individual unpaired fins (Fig. 6A, Fig. S1), perhaps due to reduced statistical power. Noteworthy exceptions are consistent correlations of *foxd3* and *egr2* with five and two network genes, respectively.

## Discussion

The identification of gene regulatory networks (GRNs) represents an exciting research avenue in the exploration of phenotypic variation. Based on resources established in model species, we can now investigate changes in GRNs in relation to the tremendous morphological diversity across non-model organisms and track down the molecular mechanisms behind morphological diversification^27–29^. In non-model teleost fishes, for instance, recent efforts targeted GRNs involved in the morphological variation of different skeletal structures^30–33^. The present study addresses the regulation of fin shape and draws upon correlated expression patterns and predicted regulatory interactions to identify members of a GRN associated with the elongated fin filaments displayed by the East African “Princess cichlid”, *N. brichardi*.

In a previous gene expression study of fin growth and shape in *N. brichardi*, we identified three genes *cx43*, *mmp9* and *sema3d* that were differentially expressed between short and long regions in both intact and regenerating unpaired fins. As explained in the introduction, the expression patterns of these genes in *N. brichardi* were not consistent with inferences on their regulatory interactions in zebrafish^18^. In particular, our data raised the possibility that *sema3d* is either regulated independently from *cx43* and *mmp9* in *N. brichardi*, or that its expression in the context of a shared network is modulated by additional regulators. Furthermore, the expression of *cx43* was positively correlated with *mmp9* expression in *N. brichardi* fins^15^, whereas reduced expression of *cx43* was associated with up-regulation of *mmp9* in the zebrafish caudal fin^20^. We also note that in contrast to zebrafish, where *esco2* functions as upstream transcriptional regulator of *cx43* and *sema3d*^34^, data in *N. brichardi* did not support a regulatory link between *esco2* and these genes^15^. In order to elucidate the regulatory background of *cx43*, *mmp9* and *sema3d* and investigate the potential divergence in regulatory mechanisms between taxa, we identified a regulatory network through the assembly of co-expression modules and prediction of their upstream regulators. Furthermore, we deduced a possible scenario explaining the discrepancy of *sema3d* expression between zebrafish and *N. brichardi*, in which an identified TF, *foxd3*, could act as transcriptional activator of *cx43* and *mmp9* and repressor of *sema3d* in *N. brichardi*.

Although the results of our previous study already suggested divergence in regulatory mechanisms between zebrafish and *N. brichardi*, the lack of data in other species necessarily restricted our search for co-expressed candidate genes to the zebrafish database. This reduces the power of our approach inasmuch as genes, which are part of the GRN in *N. brichardi* but show no expression correlations in zebrafish, will not be included among the set of candidate genes. Despite this limitation, the approach proved successful and led to the identification of a module of 10 genes with correlated expression and consistent L/S expression differences, suggesting their co-regulation through shared regulator(s) during fin formation in *N. brichardi*. Notably, the co-expression based approach to candidate gene selection yielded a similar proportion of genes with consistent L/S expression differences (10 out of 38 tested genes) as we had achieved in our previous study^15^, where candidate genes were carefully selected based on their known role in fin development, morphogenesis and/or regeneration in the zebrafish (13 out of 40 tested genes). The similar success rates suggest that after exploiting existing knowledge of gene function for the selection of candidate genes, stepwise co-expression-based candidate gene selection is indeed an efficient approach to extend the set of promising candidate genes^25,35^.

Some of the genes detected in the present study have already been implicated in studies of teleost fish fin regeneration and morphogenesis (see details in Table 1). These include *anxa2a*, a member of the annexin family^36^, two angiopoietic protein encoding genes, *angptl5* and *angptl7*^11,37^, *dpysl5a*, which encodes a member of the Collapsin response mediator protein (CRMP) family^38^, and *c1qtnf5*, encoding a basement membrane component^39^. Some other members of the gene network are not directly indicated in fin regeneration but appeared to have related functions in vertebrates. For instance, *csrp1a*, encoding a member of the cysteine-rich protein family, is required for neuron regeneration capability of adult zebrafish^40^, and *txn* and *cd63* are expressed during neural regeneration in amphibians^41^. On the contrary, an orthologue of *kif5a* in mouse, encoding a member of the kinesin family, can act as an inhibitor of neural regeneration^42^. Thus, decreased L- expression of *kif5a* and increased L-expression of *csrp1a*, *txn* and *cd63* might be indicators of faster neuronal growth in the elongated fin regions.

**Table 1:** Functions and expression patterns in appendage regeneration and morphogenesis, and regulatory connections of the identified network genes in vertebrates.

Gene functions/expression patterns		Species	References
*anxa2a*	Up-regulated in regenerating fin and limb; represses histone methylation factors involved in epigenetic regulation of caudal fin regeneration	Zebrafish Xenopus	^36,77^
*angptl5*	Involved in angiogenesis and up-regulated during elongation of caudal fin	Swordtail fish	^11^
*angptl7*	Involved in angiogenesis and blastema formation in regenerating fin	Medaka fish	^37^
*dpysl5a*	Involved in neural development and up-regulated in blastema of regenerating caudal fin	Zebrafish	^38^
*c1qtnf5*	A downstream target of Aryl hydrocarbon pathway during fin regeneration, a pathway inhibiting fin regeneration	Zebrafish	^39^
*csrp1a*	Required for neuron regeneration capability	Zebrafish	^40^
*kif5a*	An inhibitor of neuron regeneration	Mouse	^42^
*txn*	Up-regulated at onset of neural regeneration and limb regeneration	Axolotl Xenopus	^41,77^
*cd63*	Up-regulated at onset of neural regeneration	Axolotl	^41^
*myc*	Up-regulated at onset of neural regeneration, and in blastema of regenerating fin, limb and lens	Zebrafish Axolotl Xenopus	^41,51–53^
*irf8*	Involved in macrophage activation; indirectly participates in cell proliferation, growth and survival during fin regeneration	Zebrafish	^55,56^
*foxd3*	Involved in repression of Wnt pathway in bone regeneration, and up-regulated in caudal fin elongation	Human Swordtail fish	^11,65^
**Regulatory connections**			
*egr2-foxd3- myc*	*foxd3* represses *egr2* expression while its expression requires *myc* transcription during neural crest formation	Xenopus	^78,79^
*egr2-myc*	Transcriptional induction of *egr2* by *myc* in apoptotic fibroblasts	Mouse	^80^
*myc-irf8*	Antagonizing *myc* mediated transcriptional repression by *irf8* in activated macrophage	Mouse	^50^
*foxc1-foxp1*	Co-regulation of cardiac muscle differentiation and transcriptional regulation of *foxp1* by *foxc1* in hair follicle stem cells	Mouse Human	^81–83^

Six TFs with binding sites in the promoter sequences of the network genes displayed L/S differential expression consistent with the module genes, which suggests their potential cooperative transcriptional regulation of the network genes during fin morphogenesis. Three of the TFs, *foxc1*, *foxp1* and *foxd3* belong to a conserved Fork head (Fox) protein family which acts as activators or repressors in various ontogenetic processes such as developmental patterning and organogenesis in vertebrates^43,44^. The fourth TF, *myc* (*c-Myc*), encodes a nuclear phosphoprotein with diverse cellular functions which is well known for its role in the reprograming of differentiated cell types into pluripotent stem cells^45^. The fifth TF, *egr2* (*Krox20*), is a C2H2-type zinc-finger protein with prominent roles in hindbrain development^46^ and several physiological processes like bone remodelling related functions^47–49^. Finally, *irf8* (*ICSBP*) is a member of the interferon regulatory factor (IRF) family, which has a primary role in innate immunity related functions such as macrophage activation^50^. Regulatory interconnections between some of these TFs have already been demonstrated in other vertebrates (see details in Table 1). Data from the literature together with the expression correlations observed in this study, including the positive correlations between *foxc1-foxp1*, *egr2-myc*, and *myc-foxd3*, raise the possibility that one (or more) of these TFs might act upstream of the other TFs during fin formation.

Furthermore, three of these TFs, *myc*, *irf8* and *foxd3* have been already implicated in teleost fish fin regeneration/formation (Table 1). *myc* is among a few TFs required for the induction of pluripotent stem cells that appeared to be expressed during fin regeneration^51^. In amphibians, differential regulation of *myc* is also reported during blastema formation in regenerating limbs and lens^52,53^. The function of *myc* during vertebrate appendage regeneration requires further investigation but may be similar to that of its ortholog in fruit fly, which potentiates regenerative growth by abrogation of cell fate commitment in regenerating wing discs^54^. In zebrafish larva, the knock down of *irf8* causes depletion of macrophages in regenerating fins and reduces cell proliferation and growth of the regenerating fin^55^. It also leads to aberrant apoptosis of the regenerative cells, suggesting that macrophages support the survival of regenerative cells^56^. In adult zebrafish, macrophages control fin outgrowth and bony ray patterning through modulation of blastema proliferation in a stage-dependent manner^57^. The *irf8* mediated activity of macrophages might affect fin regeneration through the regulation of other potential players, *e.g. myc* and *mmp9*^58^ or/and crosstalk with Wnt/β-catenin signaling^57^, as already characterized in mammalian cells^59,60^.

Perhaps the most intriguing TF identified in our study is *foxd3*, which showed consistently higher L-expression in all fins and stages as well as expression correlations with several of the network genes across all fins. The function of *foxd3* is well studied because of its critical role in epithelial to mesenchymal transition (EMT) of the neural crest progenitors and in maintaining their multipotency (in concert with other TFs like *myc*)^61^. These processes are accompanied by fine-tuned expression regulation of downstream effectors including *cx43* and *mmp* genes^61^. Processes reminiscent of EMT and dedifferentiation of skeletal cells play a pivotal role in the regeneration of zebrafish fins^1,62^. At the molecular level, opposing activities of Wnt and BMP signaling pathways coordinate the maintenance of dedifferentiated osteoblast progenitors at the distal tip of the regenerative blastema^63^. *foxd3* is known to be a downstream effector of Wnt signaling in zebrafish^64^, and recently, a study of bone regeneration in human has demonstrated *foxd3* dependent repression of Wnt signaling pathway^65^. In addition, *foxd3* is required for the modulation of the balance between BMP and Wnt signals in developing neural crest derivatives in zebrafish^66^. Interestingly, *foxd3* is also highly expressed in the exaggerated fin outgrowth (the sword) of male sword-tail fish^11^. In our previous study, we found differential L/S expression of Wnt and BMP components in the original and regenerating fins of *N. brichardi*^15^. The above observations together with our results suggest a potential modulatory function of *foxd3* during fin elongation in *N. brichardi* by transcriptional regulation of the identified gene network and possible coordination of signals mediated by Wnt and BMP pathways (Fig. 6B).

Intriguingly, the regulatory role of *foxd3* might offer an explanation for the opposing L/S expression differences of *cx43* and *sema3d* in *N. brichardi*. Supported by findings in zebrafish, two regulatory mechanisms are possible. First, while foxd3 induces *cx43* expression in the elongated fin regions it might also repress *sema3d* expression indirectly through inhibition of Wnt pathway. Expression levels of *sema3d* and *wnt5b* were positively correlated in the fins of *N. brichardi*^15^ and Wnt has been shown to regulate *sema3d* in zebrafish^21^. Alternatively, foxd3 might act as a direct transcriptional repressor of *sema3d*, given that we found a foxd3 binding motif in the promoter sequence of *sema3d*. Indeed, a recent study demonstrated bimodal transcriptional activity of foxd3 (i.e. as transcriptional activator and repressor) and suggested *sema3d* as one of its downstream targets in zebrafish neural crest cells^67^. Figure 6B summarizes the proposed interactions between the identified genes and signalling pathways, which may underlie the regional elongation of unpaired fins in *N. brichardi*. Further functional studies are now required to confirm this regulatory mechanism during fin formation and regeneration.

## Conclusions

In the present work, we linked independent findings in vertebrates (mainly fish) in order to deduce potential regulatory interactions among co-expressed candidate genes and predicted transcription factors in the framework of a gene regulatory network associated with teleost fin shape. Some of the involved genes are known to produce signalling proteins, transcription factors or structural proteins with functions in fin formation and regeneration, whereas the role of some other genes in fin shape formation is not yet evident. Functional studies are necessary to confirm the morphogenetic impact of network genes, and comparative studies across species with similar as well as contrasting fin shapes will inform on the relationship of the identified network genes with fin shape variation.

## Methods

### Fin sampling, RNA isolation and cDNA synthesis

The tissue samples used in this study were the same as in our previous study of candidate gene analysis where fish husbandry, tissue sampling and RNA analysis protocols are described in details^15^. In brief, samples were taken from 24 captive bred adult individuals of *N. brichardi*, 12 males and 12 females with total length of 5–7 cm. Prior to fin dissection, fish were anesthetized using 0.04 gram of MS-222 per litre of water and their fins were cut in front of the first ray bifurcation (branching) under a stereomicroscope (red dashed lines in Fig. 1A,B). Tissues from the elongated and the short region of each fin biopsy (green and yellow areas in Fig. 1A) were obtained and stored frozen in RNAlater (Qiagen) until RNA isolation.

Gene expression was quantified in the original tissue (stage 0) and twice during regeneration, including a biopsy at day 15 after the first cut, when the elongated fin tips become apparent (stage 1), and another biopsy at day 35 after the second cut, when fin elongation was near to its original size (stage 2; Fig. 1B). Corresponding tissue samples from 8 fish (4 males and 4 females) were pooled as biological replicates (n = 3 replicates), and RNA isolation and cDNA synthesis was performed as described in our previous study^15^. In this paper, the tissues types are identified by fin type (dorsal, caudal, anal), region (L = elongated, S = short) and stage (0, 1, 2); for instance, aS-0 indicates the short region of the anal fin at stage 0. In the caudal fin, the dorsal and the ventral elongated regions are specified by ‘d’ and ‘v’, respectively, such that dcL and vcL refer to the dorsal and ventral elongated regions of the caudal fin, respectively (Fig. 1A). Anaesthesia and fin biopsies were performed under permit number BMWFW-66.007/0024-wF/v/3b/2016 issued by the Federal Ministry of Science, Research and Economy of Austria (BMWFW). All methods were performed in accordance with the relevant guidelines and regulations of BMWFW.

### Gene selection, Primer design and real-time qPCR

We performed a stepwise approach^25,35^ based on co-expression data available for zebrafish, COXPRESdb (http://coxpresdb.jp/) version 6.0^26^, to select candidate genes co-expressed with *mmp9*, *cx43* and *sema3d* (see Results). To attain a high degree of reliability, we filtered the genes co-expressed with each of the three genes by setting the Supportability score to a minimum of 1 (as described by Obayashi & Kinoshita^26^) (Supplementary data 1). The two rounds of candidate gene selection prompted the analysis of 39 genes, which were tested for differential expression between L and S fin tissue. In order to predict the potential upstream regulators for genes that showed L > S expression differences, we performed motif enrichment on 4 kb upstream sequences (promoter and 5′-UTR) of these genes using the annotated genome of the Nile tilapia^68^ and two algorithms: MEME^69^ and XXmotif^70^. The motifs that were present in the promoters of at least half of these genes were compared to position weight matrices (PWMs) from the TRANSFAC database^71^ using STAMP^72^ to identify matching transcription factor (TF) binding sites (Supplementary data 1).

We designed the qPCR primers for candidate genes and TFs using transcriptome data of *Neolamprologus brichardi*^73^. The 1-to-1 orthologues were confirmed by blasting zebrafish mRNA REfSeq IDs against *N. brichardi* transcriptome in NCBI and cross-checking the top hits returned by BLAST in the Ensembl database for zebrafish and Nile Tilapia orthologues (http://www.ensembl.org). The exon/exon junctions for each gene were also deduced from the Nile Tilapia annotated genome in the Ensembl database. This enabled us to design primers on exon junctions using Primer Express 3.0 software (Applied Biosystems, Foster City, CA, USA). Primers were tested for self-annealing, hetero-dimers and hairpin structures with OligoAnalyzer 3.1 (Integrated DNA Technology) (Supplementary data 3). The qPCR was conducted using Maxima SYBR Green/ROX qPCR Master Mix (2×) by following the manufacturer’s instruction (Thermo Fisher Scientific, St Leon-Rot, Germany) in 96 well-PCR plates on an ABI 7500 real-time PCR System (Applied Biosystems). The experimental set-up per run followed the preferred sample maximization method^74^ and the qPCR runs (including a dissociation step). The primer efficiency analyses in LinRegPCR v11.0 (http://LinRegPCR.nl)^75^ were conducted as described in our previous study^15^ and the efficiencies were between 89-111(E %).

### Data analysis

The mean Cq values of the two previously validated reference genes, *actb1* and *rps18*^15^, was used as Cq _reference_ and the difference between Cq values (ΔCq) of the target genes and the selected reference gene was calculated for each target gene; ΔCq _target_ = Cq _target_ − Cq _reference_. An arbitrarily selected biological replicate of dL-0, aL-0 and dcL-0 was used as calibrator sample for the dorsal, anal and caudal fin, respectively. Hence, samples were normalized to the ΔCq value of the calibrator sample (ΔCq _target_ − ΔCq _calibrator_) to obtain a ΔΔCq value. Relative expression quantities (RQ) were calculated as E^−ΔΔCq^ with E = 2^76^. For each target gene, differences in expression levels (log-transformed RQ data) between L and S tissue, fins and developmental stages were tested in a mixed linear model with biological replicate as grouping factor (Supplementary data 2). In order to identify genes with consistent L/S expression differences across fins and developmental stages, paired t-tests were performed on log-transformed RQ data. To examine expression pattern similarities between the target genes, Pearson correlation coefficients (*r*) were calculated using R (http://www.r-project.org).

### Ethical approval

All experimental protocols related to the fishes used in this study were approved by the Federal Ministry of Science, Research and Economy of Austria. Please identify the approving body and license numbers in the methods section.

### Data availability

All the data represented in this study are provided within the main manuscript or in the supplementary materials.

## Electronic supplementary material

Supplementary Figure 1.

Supplementary data 1.

Supplementary data 2.

Supplementary data 3.
