# Supplementary material for: Towards a gene regulatory network shaping the fins of the Princess cichlid
Source: Sci Rep. 2018 Jun 25;8:9602. doi: 10.1038/s41598-018-27977-y (PMC6018552; doi:10.1038/s41598-018-27977-y)
Supplement: Supplementary file 1 — Supplementary Figure 1. [file 41598_2018_27977_MOESM1_ESM.pdf]

Towards a gene regulatory network shaping the fins of the Princess cichlid

Ehsan Pashay Ahi[1],
Email: ehsan.pashay-ahi@uni-graz.at

Kristina M. Sefc[1],
Email: kristina.sefc@uni-graz.at

1. Institute of Biology, University of Graz, Universitätsplatz 2, A-8010 Graz, Austria

Corresponding Author: Ehsan Pashay Ahi,
Email: ehsan.pashay-ahi@uni-graz.at

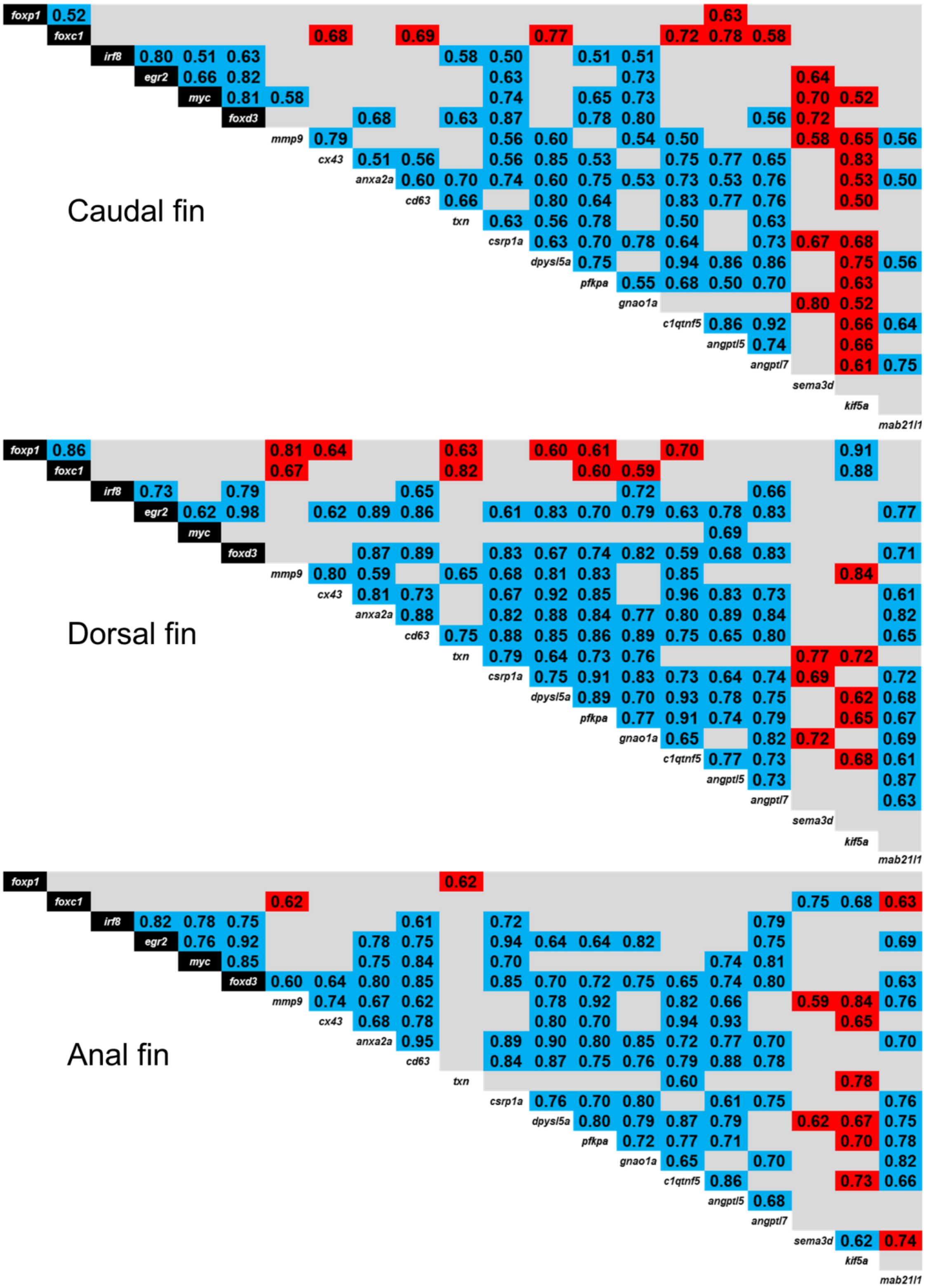

Supplementary figure 1. Significant expression correlations between members of a gene network and their predicted upstream regulators in three unpaired fins of *N. brichardi*. Numbers indicate Pearson correlation coefficients (P < 0.01 in 2-tailed tests). Blue shading represents positive and red shading represents negative expression correlation.
